# Cholinergic neurons—keeping *check on* amyloid β in the cerebral cortex

**DOI:** 10.3389/fncel.2013.00252

**Published:** 2013-12-11

**Authors:** Saak V. Ovsepian, Jochen Herms

**Affiliations:** ^1^Department of Translational Brain Research, German Center for Neurodegenerative Diseases (DZNE)Munich, Germany; ^2^Center for Neuropathology and Prion Research, Ludwig Maximilian UniversityMunich, Germany; ^3^Munich Cluster for Systems Neurology (SyNerg)Munich, Germany; ^4^Faculty of Science and Health, School of Biotechnology, Dublin City UniversityDublin, Ireland

**Keywords:** neurodegenerative disorders, Alzheimer's disease, p75 neurotrophin receptor, *basal* forebrain cholinergic neurons, amyloid β

## Abstract

The physiological relevance of p75 neurotrophin receptor-mediated internalization of ligands with no apparent trophic functions by nerve cells remains unclear. Herein, we propose a homeostatic role for this in clearance of amyloid β (Aβ) in the brain. We hypothesize that uptake of Aβ in conjunction with p75NTR followed by its degradation in lysosomes endows cholinergic basalo-cortical projections enriched in this receptor a capacity for maintaining physiological levels of this peptide in target areas. Thus, in addition to the diffuse modulator influence and channeling of extra-thalamic signals, cholinergic innervations could supply the cerebral cortex with an elaborate system for Aβ drainage. Interpreting the emerging relationship of molecular data with recognized role of cholinergic modulator system in regulating cortical activity should provide new insights into the brain physiology and mechanisms of neuro-degenerative diseases.

Depositions of Aβ plaques and neuro-fibrillary tangles in limbic, para-limbic, and associative cortices with depletion of acetylcholine (ACh) have been recognized as reliable pathological hallmarks of Alzheimer's disease (AD) (Davies and Maloney, 1976; Mesulam, 2004). Discovery of the functional relationship between the cognitive decline and loss of cholinergic markers in the plaque laden cerebral cortex with degeneration of source neurons in the nucleus basalis Meynert (NBM) *of basal forebrain* (*BF*) marked a major breakthrough in interpreting the AD since its first account in 1907 by Alzheimer (1907). Indeed, closure was reached in the 1970s of the descriptive “anamneses morbid” and a transmitter-based *hypothesis* of AD was launched, with hopeful therapeutic projections. Alas, both the functional vision and optimistic curative forecasts were doomed to *defeat*, with in-depth research revealing an incredibly composite nature of the pathology, gradually shifting the heuristic spotlight back onto descriptive grounds and focusing the main emphasis on plaque and tangle related processes (Selkoe, 1997; Holtzman et al., 2011). Thus, the significance of cholinergic deficiency in the patho-physiology of the disease was relegated to the secondary rank of undecided importance. Without doubt, such dialectical back-tracking owes itself to tough questions being identified yet *not* addressed *explicitly* by the cholinergic hypothesis (Francis et al., 1999; Terry and Buccafusco, 2003). Indeed, neither the cellular-molecular basis for the greater vulnerability of *cholinergic* axons nor the partial restorations of mnemonic and cognitive functions by anti-cholinesterase drugs have been mechanistically explained. Conceivably, most challenging to the cholinergic theory of AD were reports doubting the selective *loss* of cholinergic axons as well as the causal relationship between the degeneration of *neurons supplying ACh to the cerebral mantle* with plaque- or tangle-associated pathology (Davis et al., 1999; Zarow et al., 2003). Along with overtly intact brainstem and striatal cholinergic neurons in the AD brain with absence of amyloid plaque and neurofibrillary tangle related pathology in subjects affected by atrophy of hind-brain cholinergic nuclei, these unsettled views suggested important unknowns in the biology of the forebrain cholinergic system, in all likelihood, extending *its functions* beyond the mere supply of ACh to the cerebral cortex.

What is *unique* about *BF* cholinergic neurons and why controversy persists over their significance in the *patho-biology* of AD for over *almost a half* of a century? In addition to being one of the largest neurons in the forebrain, which channel the rostral stream of signals from the reticular core and deep brain nuclei to the cerebral mantle (extra-thalamic route), these represent the only population of nerve cells in the adult forebrain that expresses unusually high level of the p75 neurotrophin receptor (p75NTR) (Hartig et al., 1998; Mufson et al., 2008). Like other members of the tumor necrosis factor (*TNF*) receptor family to which it belongs, p75NTR lacks endogenous catalytic activity and relies on the recruitment of *co-receptors and signaling molecular partners* for initiating the cellular response. Distinctly, however, p75NTR is the only member of this family that binds neurotrophins and brain-derived growth factors, playing a key role in activation of survival or apoptotic processes (Costantini et al., 2005; Coulson et al., 2009; Knowles et al., 2009) (Figure 1). To make matters more *complex*, p75NTR also binds with high affinity to a range of collateral ligands of no obvious neurotrophic function, *including* tetanus toxins, some viral glycoproteins, prion protein and Aβ *peptide* (Yaar et al., 1997; Dechant and Barde, 2002). Although the fate of ligands bound and internalized in complex with p75NTR is a matter of *ongoing research*, emerging evidence suggests at least three routes that can be pursued by the endocytosed Aβ (Bronfman and Fainzilber, 2004; Trajkovic et al., 2008; Sorkin and von Zastrow, 2009): (1) advancement via early endosomes and trans-Golgi networks into recycling compartments with *partial* back-fusion to surface membranes; (2) formation of signaling endosomes to influence nuclear function and gene expression and (3) maturation into late endosomes destined to fusion with lysosomes and degradation of cargo or *escape through sorting in MVBs* and release in association with exosomes (Figure 1). Due to such *special* arrangements, the unusually high expression of p75NTR in BF cholinergic *neurons* is likely to render the later particularly responsive to a range of putative ligands, including Aβ (Counts and Mufson, 2005; Coulson et al., 2009; Knowles et al., 2009). On the other hand, the capacity to sequestrate and degrade Aβ by cholinergic neurons and their projections is expected to play a pivotal role in the maintenance of low physiological levels of Aβ in axon terminal fields. This intuitive notion received experimental backing from recent studies in *primary neuronal cultures of* BF, which demonstrated robust *internalization* and transport of fluor labeled Aβ in conjunction with a p75NTR antibody (IgG192-Cy3) in cholinergic neurons, with its accumulation in lysosomes (Ovsepian and Herms, 2013; Ovsepian et al., 2013). Unlike regulated endocytosis which is reliant on high voltage gated Ca^2+^ influx and canonical neuronal SNAREs (e.g., SNAP-25, syntaxin 1/2 and VAMP 1/2), the internalization of p75NTR requires Ca^2+^ entry via low-threshold T-type channels or mobilization of Ca^2+^ from thapsigargin-sensitive internal stores and is independent of VAMP 1/2 and SNAP-25. Quantitative analysis of the distribution of IgG192-Cy3 revealed its deposition in acidifying late endosomes and lysosomes—key organelles involved in degradation of cellular debris and metabolites. Although limited data is available on the relevance of these processes to Aβ clearance within the intact brain, injection of IgG192-Cy3 into the medial frontal cortex or lateral cerebral ventricle in rats also revealed its rapid axonal internalization followed by retrograde transport and accumulation in putative lysosomes of cholinergic neurons in the BF (Hartig et al., 1998; Ovsepian et al., 2013).

**Figure:**
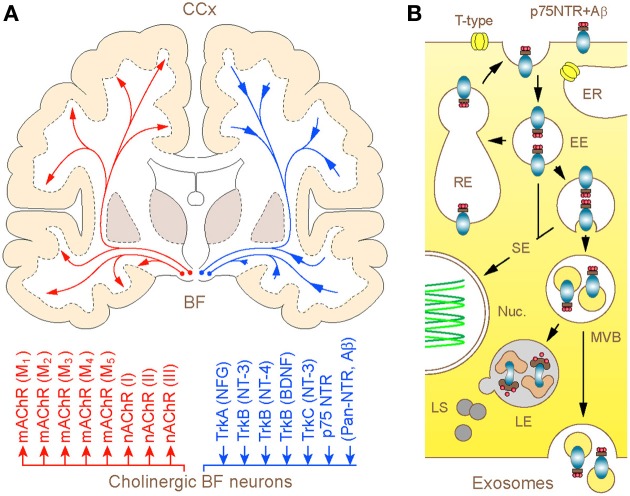
**(A)** Schematic illustration of the human BF cholinergic projections with modulator and neurotrophic mechanisms: coronal brain section. Long-range cholinergic axons and collaterals supply the entire cerebral mantle with acetylcholine (upper), which via muscarinic (mAChR1–5) and nicotinic (nAChRI-III) cholinergic receptors regulate a wide range of cortical processes and functions (lower). The diffuse projections through retrograde axonal transport channel back to the basal forebrain cholinergic neurons signaling endosomes and organelles, which carry trophic factors and other ligands bound to trkA-C and p75NTR. The homeostatic significance of p75NTR-mediated uptake of Aβ and its degradation in lysosomes of basal forebrain cholinergic neurons is discussed. **(B)** Graphical illustration of 3 possible intra-cellular routes taken by p75NTR (and Aβ) carrying endosomes. After internalization via clathrin-dependent and -independent processes, a fraction of p75NTR (and Aβ) loaded endosomes is recycled back to the plasma membrane, whereas the rest is sorted to signaling endosomes and multivesicular bodies (MVBs). From here, bulk of the cargo is degraded in hybrid MVB—lysosomal compartments, which comprise the primary metabolic hot spots of Aβ proteolysis (Mullins and Bonifacino, 2001) while a minute fraction escapes from degradation through MVB sorting into exosomes and becomes available for exocytotic released from the cell (Rajendran et al., 2006; Trajkovic et al., 2008). Abbreviations: EE and RE—early and recycling endosomes, respectively; ER—endoplasmic reticulum; SE—signaling endosomes (SE); Nuc.—nucleus; LE and LS—late endosomes and lysosomes, respectively.

Thus, it emerges that in addition to modulator functions reliant on synaptic release of ACh (Figure 1) and muscarinic (M_1_ and M_3_) receptor-mediated regulation of the processing of amyloid precursor protein (APP) (Nitsch et al., 1993; Fisher, 2012), diffuse cholinergic innervations of the cerebral cortex may also contribute to Aβ clearance. While further research is warranted to establish the contribution of the latter process to the multifarious mechanisms of *regulation of A*β *homeostasis*, direct evidence for dissociation of cognitive and anti-amyloidogenic functions of the BF cholinergic system has been provided recently (Wang et al., 2011; Laursen et al., 2013). Indeed, unlike the *selective* lesion of cholinergic cells with p75NTR targeting neurotoxin (IgG192-saporin) in AD APPswe/PS1dE9 mice, exhibiting cognitive deficit and enhanced deposition of Aβ in several cortical regions, genetic deletion of p75NTR without ablation of cholinergic neurons led to enhanced cortical Aβ loading in the absence of cognitive deficit. Together with clinical data demonstrating that neither Aβ accumulation nor cognitive deficit in AD can be attributed exclusively to depletion of cortical ACh, these findings strongly support the direct involvement of p75NTR rich cholinergic axons in clearance of cortical Aβ. Finally, the dual neuro-modulator and homeostatic *functions* of cholinergic projections received experimental support from studies of AD brain autopsies, which highlighted considerable topographic overlap between the cortical areas undergoing extensive *loss of* cholinergic *axons* and those with Aβ load (Davies and Maloney, 1976; Geula and Mesulam, 1996). Noteworthy, diffuse cholinergic projections with a large fraction of terminal *varicosities* lacking post-synaptic specializations (Descarries et al., 1997), appear highly *suited* for effective sequestration and removal of Aβ from target fields.

It is now over a century since the German psychiatrist Alois Alzheimer presented the results of the first case study of a then obscure brain disorder at the local meeting of neurologists and psychiatrists in Tübingen. The disease, according to Alzheimer, manifests in a variety of symptoms affecting first the “memory and judgment, emotion and will” and with time “the power of observation becomes blunted, old memories and experiences no longer resonate… and nothing remains of the earlier personality” (Maurer and Maurer, 1998). Although this first report failed to capture the interest of the scientific community of those days and soon fell into oblivion, the case presented then heralded *the rise of* one of the most prevalent and devastating neurodegenerative disorders of the modern age, which relentlessly crumbles all that is humane in millions affected worldwide. *In spite of* the considerable advances and tough lessons since the beginning of the 20th century, the *rising need in* effective therapies against AD dwells as blatant testimony to the shortage of knowledge and understanding of arguably one of the greatest puzzles of the Universe residing behind our eyes—the human brain.

## Conflict of interest statement

The authors declare that the research was conducted in the absence of any commercial or financial relationships that could be construed as a potential conflict of interest.
